# Mechanisms for Regulatory Effects of Exercise on Metabolic Diseases from the Lactate–Lactylation Perspective

**DOI:** 10.3390/ijms26083469

**Published:** 2025-04-08

**Authors:** Guannan Chen, Jinchao Liu, Yilan Guo, Peng Sun

**Affiliations:** 1 College of Physical Education and Health, East China Normal University, Shanghai 200241, China; 17853701690@163.com (G.C.); 10221300412@stu.ecnu.edu.cn (J.L.); yilanguo1997@163.com (Y.G.); 2 The Key Laboratory of Adolescent Health Assessment and Exercise Intervention of the Ministry of Education, East China Normal University, Shanghai 200241, China

**Keywords:** lactate, lactylation, metabolic reprogramming, insulin resistance, exercise therapy, epigenetic regulation

## Abstract

Metabolic diseases, including cardiovascular diseases, type 2 diabetes mellitus (T2DM), osteoporosis, and non-alcoholic fatty liver disease (NAFLD), constitute a major global health burden associated with chronic morbidity and mortality. Lactate, once considered as a metabolic byproduct, has emerged as a key regulator of cellular reprogramming through lactylation, a novel post-translational modification (PTM) that dynamically couples metabolic flux to chromatin remodeling. Lactylation exerts dual regulatory roles as a signaling molecule via GPR81/GPR4-mediated pathways and as a substrate for the covalent modification of histones and metabolic enzymes. Pathologically, chronic hyperlactatemia suppresses mitochondrial biogenesis, driving metabolic cardiomyopathy through the epigenetic silencing of oxidative metabolism genes. Conversely, exercise-induced lactate surges transiently enhance insulin sensitivity via *AMPK*/*PGC-1α*/*GLUT4* signaling, resolve inflammation through GPR81-mediated M2 macrophage polarization, and restore mitochondrial function via lactylation-dependent pathways. This review delineates lactylation as a spatiotemporal rheostat: chronic dysregulation perpetuates metabolic disorders, whereas acute exercise-mediated lactylation remodels transcriptional networks to restore metabolic homeostasis. Future research should integrate multiomics to clarify lactylation’s spatiotemporal dynamics, tissue-specific thresholds, metabolism–immunity interactions, and metabolic–epigenetic crosstalk for the precision management of metabolic diseases.

## 1. Introduction

The escalating global burden of metabolic diseases—including cardiovascular disorders, T2DM, obesity, and NAFLD—poses a critical public health challenge, with prevalence doubling between 1990 and 2020 [1]. Although pharmacological interventions remain central to disease management, emerging evidence underscores the pivotal roles of metabolic reprogramming and epigenetic regulation in disease pathogenesis. Among these mechanisms, lactate, once dismissed as a mere glycolytic byproduct, has emerged as a pleiotropic signaling molecule that coordinates systemic energy homeostasis through receptor-mediated pathways and a novel PTM: lysine lactylation (Kla) [2,3].

Lactate’s dual role as a metabolic intermediate and a driver of epigenetic reprogramming via lactylation positions it at the nexus of immunometabolic crosstalk. Beyond its role in redox balancing, lactate drives transcriptional and functional reprogramming via lactylation, which dynamically modifies histones (e.g., H3K18la) and nonhistone substrates (e.g., *PKM2* and *hypoxia-inducible factor 1-alpha* (*HIF-1α*)) to regulate inflammation, mitochondrial plasticity, and cellular differentiation [3,4,5]. Similarly, lactate–G-protein-coupled receptor (GPCR) signaling (e.g., GPR81/GPR4) modulates macrophage polarization, T cell activation, and endothelial function, with concentration-dependent effects on disease progression [6,7]. Paradoxically, physiological lactate gradients (<4.5 mM) support tissue repair and angiogenesis, whereas chronic hyperlactatemia (>5 mM) exacerbates insulin resistance and vascular remodeling, underscoring its context-dependent dual roles [8,9].

Exercise, a cornerstone of non-pharmacological intervention, remodels lactate flux and lactylation dynamics to counteract metabolic dysregulation. Transient exercise-induced hyperlactatemia (8–12 mM) enhances insulin sensitivity via *AMPK*/*PGC-1α*/*GLUT4* signaling, resolves chronic inflammation through GPR81-mediated macrophage M2 polarization, and restores mitochondrial oxidative capacity [10,11]. Conversely, chronic lactate accumulation in sedentary states perpetuates pathological lactylation patterns, such as H3K18la-driven PD-1 upregulation in tumor-infiltrating lymphocytes and monocarboxylate transporter 1 (MCT1)-dependent adipocyte apoptosis [5,12]. Despite these advances, critical gaps persist in elucidating how spatiotemporal lactylation dynamics orchestrate exercise-induced metabolic adaptations and disease-specific transcriptional reprogramming, particularly in tissue-specific contexts.

This review synthesizes recent breakthroughs in lactylation biology, focusing on its mechanistic roles in glucose/lipid metabolisms, immunity regulation, and tissue remodeling across cardiometabolic disorders. We further explore exercise-mediated lactate–lactylation networks as a therapeutic axis, emphasizing their potential to rewire transcriptional landscapes and mitigate metabolic inflexibility. By delineating the interplay among lactate dynamics, epigenetic reprogramming, and exercise physiology, this work aims to bridge translational gaps and identify precision-targeted strategies for metabolic syndrome management.

## 2. Overview of Lactate and Metabolic Diseases

Lactate, traditionally viewed as a glycolytic byproduct, has emerged as a central hub coordinating metabolic adaptation across physiological systems [13]. Although oxidative metabolism predominantly directs pyruvate into the mitochondrial tricarboxylic acid cycle in normoxia, hypoxic stress or mitochondrial insufficiency triggers LDHA-mediated lactate generation—a process now recognized as integral to cellular redox balancing and epigenetic regulation via lactylation [14].

The skeletal muscle–liver lactate shuttle exemplifies systemic metabolic integration. High-intensity exercise induces transient muscular lactate accumulation via anaerobic glycolysis (generating ATP 100-fold faster than in oxidative phosphorylation), while endurance training enhances hepatic Cori cycle activity—converting lactate to glucose at energy costs equivalent to six ATPs per molecule recycled [2].

Within the central nervous system (CNS), the astrocyte–neuron lactate shuttle (ANLS) operates as a neurovascular coupling mechanism [15]. Cognitive activation triggers astrocytic glycogenolysis, producing lactate, which fuels neuronal long-term potentiation via monocarboxylate transporter 2 (MCT2)-dependent uptake [16]. Recent studies have revealed that this metabolic coupling extends to immunity regulation: microglial GPR81 receptors sense extracellular lactate gradients, suppressing neuroinflammation through CREB-mediated anti-inflammatory cytokine production [17,18]. Notably, neuronal activity induces histone lactylation at plasticity-related gene loci [19], suggesting that lactate may directly bridge energy metabolism and epigenetic memory formation.

Gut-microbiota-derived D-lactate adds another dimension to lactate’s systemic signaling. Commensal bacteria generate stereospecific D-lactate isomers that strengthen intestinal barrier functions through complementary mechanisms: HDAC3 lactylation in colonic epithelia enhances tight junction assembly [20,21]. However, dysbiosis-induced D-lactate overproduction may paradoxically impair barrier integrity via excessive matrix metalloproteinase activation [22], illustrating the delicate balance in lactate-mediated mucosal homeostasis.

The pathophysiological duality of lactate manifests most strikingly in immunometabolic crosstalk. Physiological lactate gradients support tissue repair through *HIF-1α*/VEGF-driven angiogenesis [23] and PPARα-mediated fatty acid oxidation. Conversely, chronic lactate accumulation creates an immunosuppressive niche via two synergistic pathways: (1) M2 macrophage polarization through *PKM2* lactylation [4] and (2) PD-1 upregulation in tumor-infiltrating lymphocytes via histone H3K18 lactylation [5,6].

### 2.1. Intercellular Signaling

It has been reviewed that the lactate shuttle theory (LST) elucidates the process of lactate transport between cells, which has energy transfer and signaling functions [2]. Lactate is transported across cells via monocarboxylate transporters (MCTs), which regulate various physiological and pathological cellular processes [23]. According to Felmlee et al., MCT belongs to the solute carrier (SLC) transporter protein family, which consists of 14 different proteins (MCT1-MCT14) [24] (Table 1). These proteins facilitate the transport of monocarboxylic acids, such as lactate, pyruvate, and ketone bodies, across cell membranes [25].

In contemporary scientific discourse, lactate is recognized as a signaling molecule capable of exerting its biological functions through three major classes of G-protein-coupled receptors (GPCRs) [34]. The primary known lactate receptors include GPR81, GPR132, and the proton-sensing GPR4. GPR81 exhibits tissue-specific expression patterns, with high abundance in adipocytes, neurons, and myeloid cells, playing pivotal roles in both physiological and pathological processes [23]. Adipocyte GPR81 activation inhibits hormone-sensitive lipase (HSL) phosphorylation via cAMP/PKA signaling, thereby reducing lipolysis at physiological lactate concentrations [8]. In immunity regulation, GPR81 exerts anti-inflammatory effects by blocking IκBα degradation to prevent NF-κB nuclear translocation and disrupting ASC oligomerization required for NLRP3 inflammasome assembly [35]. GPR81 agonists suppress fasting plasma free-fatty-acid levels in rodents and improve insulin sensitivity in mouse models of insulin resistance and diabetes, highlighting its therapeutic potential [36].

GPR132 is a lactate-sensitive GPCR that plays dual roles in metabolic diseases by modulating energy metabolism and inflammatory responses [37]. This leads to the suppression of lipolysis and promotion of lipogenesis in adipocytes while driving anti-inflammatory M2 polarization in macrophages, thereby improving insulin sensitivity [37].

GPR4, a proton-sensing GPCR highly expressed in vascular endothelial cells, orchestrates vascular inflammation through pH-dependent activation cascades [38]. At pathological pH levels, lactate synergizes with protons to stabilize GPR4-β–arrestin complexes, amplifying downstream signaling in various pathological states, including inflammation and ischemia [7]. GPR4 also induces ER stress and apoptosis in endothelial cells and promotes angiogenesis by regulating endothelial cells’ tubule formation, migration, and proliferation under acidic conditions [39]. Additionally, lactate activates dendritic cells and modulates the immune response through paracrine signaling [7]. Emerging evidence reveals crosstalk between lactate receptors and epigenetic modifications. *Fam172a* modulates *POMC* neuronal activity through H4K12-lactylation-dependent chromatin remodeling at neuropeptide gene loci (*AgRP* and *POMC*), with hypothalamic H4K12la levels correlating inversely with bodyweight in diet-induced obesity models [40].

### 2.2. Lactate Regulates Inflammation and Immune Responses

Lactate has been shown to modulate the functions of immune cells via multiple pathways [41]. For instance, lactate inhibits the activation of proinflammatory macrophages and promotes their conversion to an anti-inflammatory phenotype, thereby mitigating the inflammatory response [42]. Additionally, lactate inhibits metabolic reprogramming and proinflammatory responses in macrophages via receptors such as GPR81 [43,44]. Moreover, studies have shown that lactate can inhibit T-cell overactivation, thereby exerting an immunomodulatory effect on chronic inflammation [6].

Lactate exhibits a dual role in the inflammatory microenvironment. It serves as an energy source to support the metabolic needs of immune cells and regulates gene expression and protein function via mechanisms such as lactate modification, thereby influencing the intensity and duration of the inflammatory response [3]. Moreover, lactate contributes to tissue repair by regulating immune cell functions and promoting a balance between inflammation and repair [45]. In the context of inflammation, lactate accumulation can inhibit mitochondrial function, thereby prompting a rapid shift toward glycolysis to maintain cellular energy production and ensure cellular survival and functions [45].

Lactate significantly influences disease development and therapeutic efficacy. Lactate’s roles in the microenvironments of chronic inflammatory diseases have led to novel therapeutic concepts [46]. For instance, modulating lactate metabolism and regulating lactate levels have been proposed as potential therapeutic strategies for inflammatory diseases [46].

In summary, lactate has multiple critical roles in organismal metabolism, both as a glycolysis end product and as a signaling molecule regulating intercellular signaling, inflammation, and immune responses via GPCRs. The lactate shuttle hypothesis indicates that lactate regulates cellular physiological and pathological processes via the intercellular shuttle process of MCTs. Additionally, lactate fulfills a dual role in the inflammatory microenvironment by serving as an energy source for immune cells and regulating gene expression and protein function via lactate modification, thereby impacting the intensity and duration of inflammatory responses.

### 2.3. Association of Lactate with Metabolic Diseases

Lactate exhibits concentration-dependent duality in metabolic regulation, with its pathophysiological impact determined by temporal–spatial dynamics and receptor activation thresholds.

#### 2.3.1. T2DM

Abnormal lactate levels are associated with dysregulated glycemic control, affecting insulin sensitivity [47]. In the management of T2DM, it is imperative to monitor lactate levels and adjust the intervention program accordingly. A study found that patients with T2DM exhibited slightly elevated blood lactate levels [48]. These slightly elevated lactate levels may be associated with a relative deficiency in insulin secretion, leading to decreased mitochondrial pyruvate utilization and increased glycolysis, especially when blood glucose is elevated, potentially resulting in increased blood lactate production [49]. Slightly elevated lactate levels have been shown to exacerbate insulin resistance and be detrimental to long-term glycemic control [12].

Exercise constitutes a pivotal element in the management of T2DM [30]. Exercise interventions modulate lactate metabolism through:

Acute effects: Post-exercise lactate elevation, typically reaching levels of 8–12 mM, is associated with enhanced insulin sensitivity. This occurs via the activation of the *AMPK*/*PGC-1α* pathway, which mediates the translocation of glucose transporter type 4 (*GLUT4*) [50,51]. AMP-activated protein kinase (*AMPK*), an energy-sensing enzyme, is activated in response to exercise-induced metabolic stress, including increases in lactate levels [52]. Activated *AMPK* then phosphorylates downstream targets [53]. *PGC-1α*, a key regulator of mitochondrial biogenesis and energy metabolism, is also regulated by *AMPK* [54]. *GLUT4* is a glucose transporter protein, and its translocation to the cell membrane allows for increased glucose uptake into cells, thereby enhancing insulin sensitivity [55].

Chronic adaptation: Regular moderate-intensity aerobic and resistance exercises have been shown to reduce blood glucose levels, decrease insulin resistance, improve lactate metabolism, and increase the body’s tolerance to lactate in patients with T2DM [56].

#### 2.3.2. Obesity

Lactate is a double-edged sword in adipose remodeling. Obesity, a complex metabolic disorder, is intricately linked to dysregulated lactate metabolism. Studies have demonstrated that adipose tissue hypoxia contributes to elevated lactate levels in obese individuals [57,58]. Dynamic fluctuations in the lactate concentration critically influence metabolic homeostasis and disease progression through multifaceted mechanisms.

Slightly elevated lactate levels (<4.5 mM) can have beneficial effects on metabolic regulation through three pathways: (1) Enhanced glucose metabolism: Lactate stimulates glucose oxidation, ameliorating glucose–lipid metabolic imbalances [57]; (2) Suppression of lipolysis: It downregulates adipose tissue lipolysis, thereby reducing free-fatty-acid release [59]; and (3) Maintenance of adipocyte homeostasis: Lactate prevents pathological adipocyte hypertrophy and functional impairment [60]. Furthermore, lactate activates the GPR81 receptor to promote extracellular efflux [61], mitigating intracellular accumulation. This mechanism inhibits adipocyte apoptotic signaling and inflammatory cytokine secretion, ultimately enhancing systemic insulin sensitivity.

At concentrations exceeding 4.5 mM, lactate paradoxically exacerbates metabolic disturbances by aggravating systemic inflammation and insulin resistance [62] and disrupting adipose tissue functionality by impairing the thermogenic and lipolytic balance [63].

Adipose tissue is the central hub of lactate homeostasis. Adipose tissue accounts for >50% of the resting-state lactate production in humans [64], establishing its pivotal role in systemic lactate dynamics. Mechanistically, the disruption of monocarboxylate transporter 1 (MCT1) in adipocytes induces intracellular lactate accumulation, triggering apoptotic cascades and proinflammatory cytokine release [12,65]. These pathological changes exacerbate obesity-associated insulin resistance, highlighting the tissue-specific regulatory importance of lactate flux and providing evidence that lactate is a key mediator of obesity-related insulin resistance [66]. The pharmacological modulation of adipose lactate transport, particularly through MCT1 or GPR81 targeting, may represent a novel therapeutic strategy for metabolic dysfunction in obesity.

Consequently, lactate plays multifaceted and pivotal roles in a broad spectrum of metabolic diseases, and fluctuations in its concentration have substantial impacts on disease progression and therapeutic outcomes. Therefore, in the management of metabolic diseases, it is imperative to monitor lactate levels and adjust intervention programs accordingly.

## 3. Mechanisms of Lactylation in Metabolic Diseases

### 3.1. Lactylation-Dependent Gene Regulation

Lactylation, a recently characterized metabolite-driven post-translational modification, regulates gene expressions through dual mechanisms.

#### 3.1.1. Direct Transcriptional Control

Pan-histone profiling may identify multiple conserved lactylation sites, with H3K18la strongly correlating with transcriptional activation [3]. Neural stem cells (NSCs) exhibit reduced neurogenesis via H4K12la-mediated *p53* stabilization, highlighting lactylation’s role in cellular plasticity [67].

#### 3.1.2. Indirect Chromatin Remodeling

Lactylation modulates transcription factors and enzymes to reshape chromatin landscapes. For example, *YY1* lactylation increases in retinal microglia, promoting the activation of microglia and transcription of inflammatory genes, thus aggravating autoimmune uveitis [68].

#### 3.1.3. Dynamic Interactions Between Lactylation and Other Epigenetic Modifications

As a newly emerging PTM, lactylation exhibits dynamic interaction mechanisms, with classical modifications, such as acetylation and methylation, in the epigenetic regulatory network. It has been found that lactylation and acetylation have highly similar distribution characteristics in the genome, sharing some modification enzyme systems (e.g., p300 and HDAC1-3) and regulating gene expressions through the competitive modification of lysine residues. In the process of liver fibrosis, *hexokinase-2* (*HK2*) inhibits the transcriptional activation of fibrosis-suppressing genes by promoting lactylation at histone H3K18 sites rather than acetylation, which reveals the competitive substitution mechanism of lactylation at acetylation modification sites [69,70]. This competitive relationship may be because of substrate bias shifts induced by changes in the metabolic microenvironment—when intracellular lactate levels rise, lactoacyl-CoA production increases, prompting the modifying enzyme to catalyze lactylation over acetylation [71]. However, the interaction between the two is significantly cell-type dependent: in anoxic HeLa cells, the lactylation level increases as the acetylation level decreases, while in LPS-activated macrophages, the two synergize to promote HMGB1 modification and exosome release [72,73], suggesting that metabolic reprogramming can restore the modification network’s balance by regulating the substrate’s availability and enzymes’ kinetic parameters.

There is a synergistic regulation between lactylation and methylation. In the process of DNA damage repair, H2BK123ub (the ubiquitination modification of histone H2B) was found to promote the methylations of H3K4, H3K46, and H3K79, which are essential for DNA damage repair. At the same time, some studies have shown that there is a correlation between lactylation and methylation such that in some cases, lactylation may affect the level of the methylation modification, but its specific mechanism needs to be further studied [74].

As the hub of the metabolism–epigenetic network, lactylation enables the dynamic coupling of the cellular metabolic state and gene expression program. During the polarization of macrophages, lactate produced by enhanced glucose metabolism drives phenotypic transition to the repair type by promoting histone lactylation in the promoter region of repair genes, such as Arg1 [72]. This metabolic signal transduction depends on the homeostasis of lactoacyl-CoA. When metabolic parameters, such as glucose and oxygen levels in the microenvironment, change, the lactylation modification level can respond sensitively and then reprogram the gene expression profile by regulating the chromatin accessibility and transcription factor activity [71,75]. It is worth noting that the hub role of the lactylation network is reflected not only in its regulation of classical PTM but also in its ability to integrate multidimensional biological information as a “metabolic sensor”; through competitive inhibition, collaborative enhancement, and other mechanisms, lactylation modifications translate metabolic flux changes into precise epigenetic instructions. Finally, the precise control of the cellular fate determination and pathophysiological processes is realized.

### 3.2. The Roles of Lactylation in Inflammation and Immune Responses

Lactylation serves as a metabolic checkpoint in inflammatory responses through dual regulatory mechanisms:

Macrophage Polarization: The lactylation of *PKM2* at K62 promotes M2 polarization by enhancing tetramer formation, resolving inflammation via anti-cytokine production [4].

T-Cell Functional Reprogramming: In rheumatoid arthritis, lactate has been shown to prolong chronic inflammation by regulating T-cell function and promoting the production of inflammatory factors [76].

Inflammatory Microenvironment Crosstalk: Collectively, the critical roles of lactate homeostasis, lactate shuttling, and lactylation in acute and chronic inflammatory responses are highlighted by the inflammatory microenvironment [45].

### 3.3. Interaction of Lactylation with Metabolic Pathways

#### 3.3.1. Lactylation and Glucose Metabolism

Lactylation establishes a bidirectional loop between glycolysis and epigenetics. Lactate-derived lactyl-CoA facilitates nuclear translocation, activating glycolytic genes (e.g., *PKM2*) to amplify the glucose flux [77]. Neuronal lactate shuttling via LDH1 sustains synaptic ATP during hypoxia, illustrating lactylation’s role in compartmentalized energy metabolism [78]. This metabolic flexibility suggests that lactylation may serve as an evolutionarily conserved mechanism for optimizing energy substrate utilization under varying oxygen tensions. Systemically, lactylation fine tunes insulin sensitivity through *PI3K*/*Akt* pathway modulation, linking cellular metabolism to whole-body glucose homeostasis [79]. Lactylation, with its dual regulatory capacity of simultaneously governing cellular metabolism and systemic responses, is positioned as a critical node in the pathogenesis of metabolic diseases.

#### 3.3.2. Lactylation and Lipid Metabolism

Lactylation synchronizes lipogenesis with glycolytic NADPH production. Hepatic *FASN* lactylation may potentially stabilize its catalytic domain and contribute to lipid droplet biogenesis in NAFLD [5,80]. Paradoxically, mitochondrial CPT1A enhances fatty acid oxidation acutely but promotes lipid storage chronically, demonstrating context-dependent regulation [81]. These opposing effects underscore lactylation’s role as a metabolic buffer system.

#### 3.3.3. Lactylation and Energy Metabolism

In hypoxia, lactylation shifts energy production from oxidative phosphorylation (OXPHOS) to glycolysis via PDH inhibition, preserving the redox balance [82]. In cardiomyocytes, acute lactylation stabilizes *HIF-1α* during ischemia, enhancing glycolytic gene expression to maintain ATP production [23]. Conversely, chronic hyperlactatemia suppresses mitochondrial biogenesis, driving metabolic cardiomyopathy through the epigenetic silencing of oxidative metabolism genes [83].

### 3.4. Tissue-Specific Lactylation Dynamics

#### 3.4.1. Liver: NASH and Liver Fibrosis

Studies have shown that lactylation can regulate the expressions of glycolysis-related genes in liver cells and affect energy metabolism in the liver [3,84,85,86]. For example, E3 ubiquitin ligase (TRIM56) slows NAFLD progression by ubiquitinating *FASN*, a key protein in fatty acid synthesis. TRIM56 is downregulated in human NAFLD liver tissue and diet-induced mouse NAFLD liver tissue, while its gene level remains unchanged, and changes in TRIM56 protein expression occur only in liver cells [87]. These findings shed light on the potential role of lactate modification in the development of NASH, possibly by regulating the transcriptions and protein translations of specific genes involved in the progression of the disease. In liver fibrosis, lactylation affects the liver structure by regulating collagen expression. Studies have shown that *HK2*-induced lactate promotes histone lactylation, which controls stellate cellular activation and leads to liver fibrosis. The stellate-cell-specific or systemic deletion of *HK2* to inhibit H3K18la can mitigate stellate cellular activation and liver fibrosis [69,70,88] (Table 2).

#### 3.4.2. Heart: Ischemic Remodeling

Lactylation plays a crucial role in enhancing post-infarction repair in patients with metabolic disorders, such as diabetes, complicated by myocardial infarction. This is achieved through histone lactylation (e.g., *VEGFA*) and macrophage modulation, which help to establish immunity homeostasis and activate the cardiac repair process in a timely manner [89] (Table 2). Exercise-induced MECP2K271la may attenuate atherosclerosis in patients with metabolic disorders by suppressing vascular adhesion molecules (e.g., ICAM-1) and promoting anti-inflammatory pathways (Table 2). This finding provides a potential therapeutic approach for metabolic-syndrome-related cardiovascular diseases [90,91,92].

#### 3.4.3. Bone: Osteoporosis

Lactate modification activates osteogenesis-related genes, including the collagen type I alpha 2 chain (COL1A2), cartilage oligomeric matrix protein (COMP), ectonucleotide pyrophosphatase/phosphodiesterase 1 (ENPP1) [93], and *transcription factor 7-like 2* (*TCF7L2*) [94]. These genes are essential for osteoblast differentiation and functions. Conversely, it inhibits osteoclastogenesis through LDHA suppression, reducing bone resorption [95,96]. Consequently, this dual regulation maintains bone homeostasis, with dysregulation contributing to osteoporosis.

**Table 2:** Lactylation sites of the key metabolic organs.

Organ	Target(s)	Mechanism(s) of Action	Reference(s)
Liver	H3K18la and H3K9la	Studies have shown that *HK2*-induced lactate promotes histone lactylation, which controls stellate cellular activation and leads to liver fibrosis. The stellate-cell-specific or systemic deletion of *HK2* to inhibit H3K18la can mitigate stellate cellular activation and liver fibrosis. The inhibition of H3K9la can inhibit HCC development.	[69,70,88]
Heart	*VEGFA*, H3K9la, and MECP2K271la	Histone lactylation (e.g., *VEGFA*) helps to establish immunity homeostasis and activate the cardiac repair process in a timely manner. A feedback loop between H3K9la and HDAC2 drives VEGF-induced angiogenesis. Exercise-induced MECP2K271la may attenuate atherosclerosis in patients with metabolic disorders by suppressing vascular adhesion molecules (e.g., ICAM-1) and promoting anti-inflammatory pathways.	[89,90,91,92]
Adipose	*APOC2*-K70la	Lactate stabilizes *APOC2* and promotes extracellular lipolysis by enhancing lactylation at the K70 site.	[97]
Brain	SNAP91	Lactate-mediated lactylation of synaptosomal SNAP91 in the prefrontal cortex enhances synaptic plasticity, mitigating anxiety-like behaviors.	[98]

**Figure 1:**
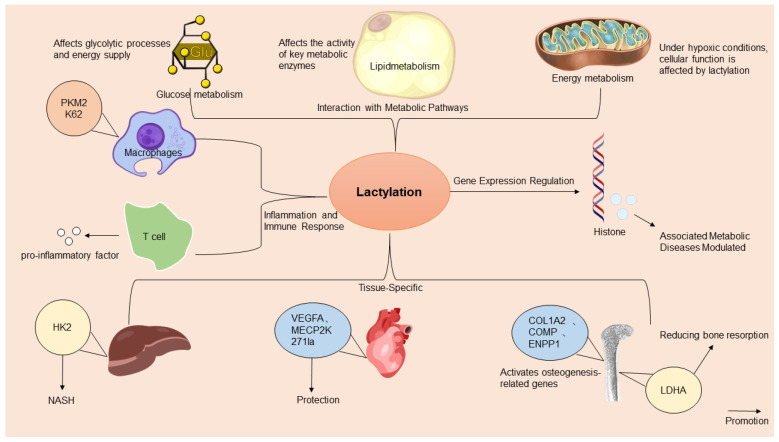
A comprehensive overview of the multifaceted mechanisms of action of lactylation in metabolic diseases. Lactylation, a significant epigenetic modification, influences metabolic diseases by regulating gene expressions, metabolic pathways, inflammation, tissue-specific lactylation dynamics, and immune responses. It directly affects gene transcription by modifying histones and transcription factors. Adapted from SciDraw (www.scidraw.io) under CC BY 4.0.

In summary, lactylation integrates metabolic flux with epigenetic and immunity regulations, acting as a spatiotemporal rheostat in metabolic diseases. Future studies should prioritize tissue-specific thresholds and the therapeutic targeting of lactylation dynamics to address disease-specific metabolic inflexibility (Figure 1).

## 4. Lactate–Lactylation-Mediated Regulation of Exercise in Metabolic Diseases

**Figure 2:**
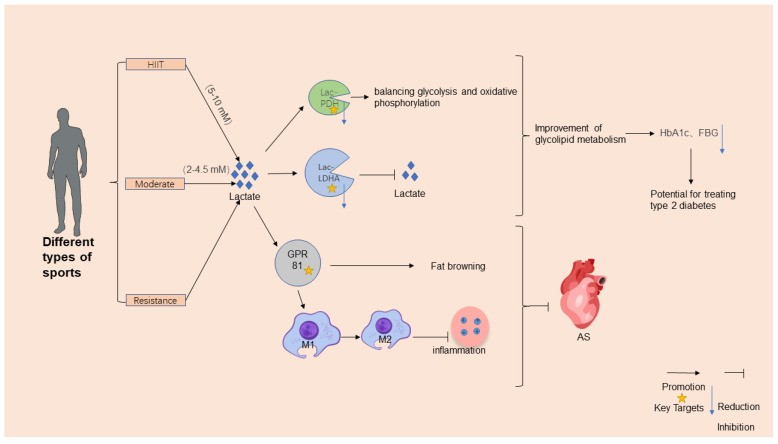
Mechanisms for exercise’s regulatory effects on metabolic diseases. Exercise positively regulates metabolic diseases by enhancing lactate metabolism and lactylation. High-intensity or hypoxic exercise increases the muscles’ production of lactate, which is converted to glucose via hepatic gluconeogenesis. Lactylation, modified by exercise, impacts glucose and energy metabolisms, influencing metabolic disease development. Exercise protects against metabolic diseases through mechanisms such as improved insulin sensitivity, enhanced lipid metabolism, reduced inflammation, and optimized mitochondrial and nervous system functions. Adapted from SciDraw (www.scidraw.io) under CC BY 4.0.

The dynamic interplay among exercise, lactate metabolism, and lactylation modifications serves as a pivotal axis in understanding exercise-induced protection against metabolic disorders. During high-intensity exercise, rapid glycolysis generates the accumulation of transient lactate (5–10 mM) [8], which functions as both a metabolic intermediate and a signaling molecule. This lactate surge activates GPR81, a lactate-sensitive receptor, to stimulate angiogenesis [99], promote adipose tissue browning [100] (Figure 2), and enhance immunity modulation by shifting macrophage polarization from proinflammatory M1 to anti-inflammatory M2 phenotypes [11,101] (Figure 2). Conversely, moderate-intensity exercise (2–4.5 mM) improves the mitochondrial oxidative capacity and reduces chronic lactate overproduction, thereby enhancing metabolic flexibility and lactate clearance. Systematic reviews highlight that regular moderate-intensity exercise lowers baseline blood lactate levels and increases the lactate threshold in T2DM patients, improving their exercise tolerance and glycemic control [102]. Critically, exercise-induced lactate dynamics regulate lactylation, where lactate-derived acyl groups modulate protein functions. Lactylation fine tunes metabolic enzymes, such as PDH and LDHA, balancing glycolysis and oxidative phosphorylation [103]; stabilizes hypoxia-inducible factor *HIF-1α* to adapt to oxygen level fluctuations [81]; and suppresses inflammatory gene transcription in adipose tissue, thereby alleviating obesity-related metabolic dysfunction [90]. 

Exercise further combats metabolic diseases through multilayered mechanisms. Aerobic exercise, particularly high-intensity interval training (HIIT), enhances insulin sensitivity by upregulating mitochondrial biogenesis and glucose transporter GLUT-4 expression [104], while moderate-intensity aerobic exercise boosts free-fatty-acid oxidation, reducing plasma triglyceride levels and improving lipid profiles [105]. Resistance training activates the *PI3K*/*Akt* pathway to enhance insulin signaling and the muscles’ glucose uptake [106] while also increasing muscle mass to elevate the basal metabolic rate and caloric expenditure [107]. Both exercise modalities reduce systemic inflammation by lowering proinflammatory cytokine levels (e.g., TNF-α and IL-6) and promoting anti-inflammatory macrophage polarization [4,108].

Notably, exercise-induced lactate not only modulates peripheral metabolism but also exerts neuroprotective effects. For instance, the lactate-mediated lactylation of synaptosomal SNAP91 in the prefrontal cortex enhances synaptic plasticity, mitigating anxiety-like behaviors [98] (Table 2), while lactate–lactylation networks regulate immunity–metabolism crosstalk in cardiovascular diseases via mitochondrial modulation [3]. However, lactate dynamics display context-dependent effects. Emerging evidence reveals that excessive exercise induces muscle-derived lactate-rich small extracellular vesicles (sEVs) containing *FBXO2* and lactylated *SORBS3* proteins. These sEVs activate hepatic stellate cells through systemic circulation, triggering pro-fibrotic transformation and inflammatory pathway activation, ultimately accelerating hepatic fibrogenesis [109]. This pathological process contrasts sharply with the transient lactylation events during moderate exercise, highlighting the importance of exercise intensity modulation.

Clinically, chronic hyperlactatemia in diabetes [110] and hypoxia [111] exacerbates insulin resistance through sustained pathological lactylation, whereas controlled exercise interventions lower HbA1c, improve lipid profiles [112], and enhance mitochondrial biogenesis [113]. The differential outcomes underscore lactate’s role as a metabolic rheostat: physiological fluctuations promote homeostasis, while chronic elevation or acute overload drives pathology. Targeting lactylation dynamics and sEV generation may, thus, provide novel therapeutic strategies for exercise-associated organ injury.

There were significant physiological/pathological differences in the dynamic characteristics and metabolic regulation of lactate modification [114]. During acute stimuli, such as exercise, a surge in lactate levels can rapidly induce lactate modification of both histone (e.g., H3K18) and non-histone (e.g., *PKM2*) proteins, with the peak appearing within 30 min and rapidly fading with prolonged lactate metabolism. This short-term modification enhances insulin sensitivity by enhancing glycolysis [90]. Conversely, long-term modifications, triggered by persistently high lactylation in chronic diseases, inhibit mitochondrial biogenesis genes, leading to mitochondrial dysfunction and exacerbating insulin resistance. The study suggests that lactate modification has a time threshold effect: the transient modification of an acute stimulus has a metabolic protective effect, while continuous modification turns to pathological processes. At present, it is necessary to analyze its dynamic regulation mechanism with multiomic technology to reveal the key nodes of metabolic homeostasis transformation.

## 5. Potential of Lactylation in the Treatment of Metabolic Diseases

### 5.1. Potential of Lactylation as a Therapeutic Target

Emerging evidence positions protein lactylation as a pivotal post-translational modification with profound implications for metabolic disease pathogenesis and therapeutic development. The dynamic regulation of histone lactylation through NSC lactate homeostasis establishes a critical interface between cellular metabolism and tissue regeneration. Mechanistically, this process modulates the MDM2-*p53* signaling axis [67], suggesting that lactylation serves as a metabolic rheostat by coordinating cellular proliferation and metabolic adaptation. This regulatory paradigm extends beyond histones, as lactylation-induced conformational changes in the chromatin architecture [115] create an epigenetic landscape that fine tunes transcriptional programs governing glucose and lipid metabolisms.

The immunometabolic dimension of lactylation reveals intriguing therapeutic possibilities. In macrophage polarization dynamics, lactate-mediated *PKM2* activation exerts dual regulatory effects: the suppression of the Warburg metabolism and induction of pro-reparative phenotypic switching [4]. This metabolic checkpoint mechanism demonstrates how lactylation coordinates inflammatory resolution through metabolic reprogramming. Notably, exercise-induced immunomodulation appears to exploit this pathway through macrophage polarization toward M2 anti-inflammatory phenotypes, suggesting endogenous mechanisms for lactylation pathway regulation [90].

The pathological implications of lactylation are evident in metabolic diseases. Abnormal lactylation in key proteins related to metabolic disorders may lead to the dysregulation of metabolic pathways and exacerbate the progression of metabolic diseases. For example, in certain cases of diabetes, the aberrant lactylation of specific proteins might contribute to the development of diabetic complications by affecting glucose metabolism and insulin signaling. Conversely, histone lactylation (e.g., *VEGFA*) and macrophage regulation help to establish immunity homeostasis and activate the cardiac repair process in a timely manner [89]. These contrasting pathophysiological roles underscore the context-dependent nature of lactylation signaling.

Therapeutic development requires addressing three key challenges: (1) the tissue-specific regulation of the lactate flux to modulate lactylation patterns, (2) the development of enzymes targeting specific lactylation sites, and (3) the temporal control of lactylation dynamics during disease progression. Current evidence suggests that combinatorial approaches targeting both lactate metabolism and post-translational modification machinery may yield synergistic therapeutic effects across metabolic, inflammatory, and cardiovascular disorders.

### 5.2. Clinical Applications of Lactylation Modifiers

The development of lactylation modifiers represents a paradigm shift in targeting lactate-centric pathologies through the dual modulation of the metabolic flux and epigenetic reprogramming. Current therapeutic strategies focus on three principal axes: (1) systemic lactate metabolism regulation, (2) site-specific lactylation intervention, and (3) lactate-receptor-signaling modulation.

**Figure 3:**
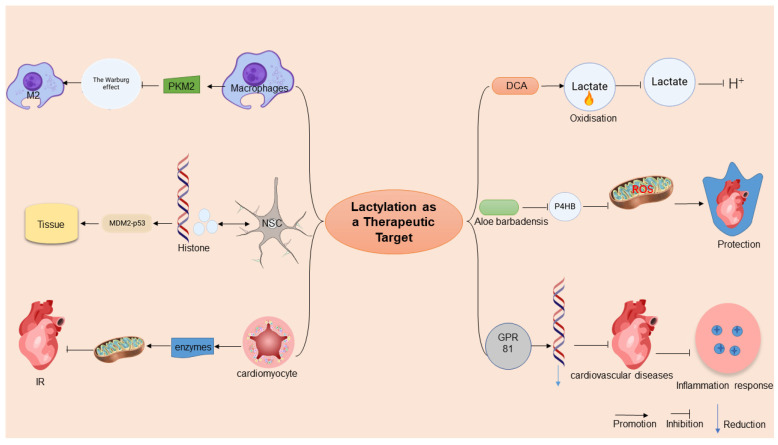
Potential of lactylation in the treatment of metabolic diseases. This figure illustrates the therapeutic potential of lactylation in metabolic diseases. Lactylation modifies histone conformations, influencing gene expressions and serving as an anti-inflammatory target by altering macrophage phenotypes and mitochondrial functions. In clinical applications, lactylation modifiers, like sodium dichloroacetate (DCA) and aloe vera rhodopsin, target lactate metabolism, reducing acidosis and myocardial injury. Adapted from SciDraw (www.scidraw.io) under CC BY 4.0.

Metabolic Homeostasis Restoration: Pharmacological lactate modulators demonstrate therapeutic efficacy by rewiring the cellular energy metabolism. As a *PDK* inhibitor, DCA can activate PDH by inhibiting *PDK2*, promoting pyruvate to enter the tricarboxylic acid cycle, and reducing lactylation accumulation, thereby improving energy metabolism disorders and oxidative stress in cerebral ischemia reperfusion (I/R) injury [116] (Figure 3). However, the specificity of its mechanism of action and potential off-target effects require careful evaluation. First of all, there are many isoenzymes in the *PDK* family (*PDK1-4*), which only emphasize the effective inhibition of DCA by *PDK2*, without clarifying its specific effects on other isoenzymes, which may lead to non-targeted effects. For example, the key role of *PDK1* in cardiometabolic functions, if inhibited, may interfere with tissue-specific metabolic balance. Second, the activation of PDH by DCA may indirectly affect the acetylation modification network. If DCA changes intracellular acetyl-CoA levels through metabolic reprogramming, it may interfere with the acetylation status of histone or non-histone proteins, thereby affecting gene expressions or protein functions. In addition, studies have confirmed that DCA exerts antioxidant effects through the *Nrf2* pathway, but whether *Nrf2* activation is entirely dependent on the *PDK2*-PDH axis is unclear. The DCA protective effect disappeared after ML385 inhibited *Nrf2*, suggesting that *Nrf2* is the core downstream target, but the molecular coupling mechanism between PDH activation and *Nrf2* signaling still needs to be further analyzed [117]. In conclusion, although DCA plays a neuroprotective role through the *PDK2*-PDH-*Nrf2* axis, its potential effects on metabolic modifications other than lactylation (e.g., acetylation) and multi-target regulation need to be further validated to ensure its therapeutic specificity and avoid off-target risks. 

Precision Targeting of Lactylation Sites: Emerging evidence highlights the therapeutic potential of direct lactylation site modulation. Aloin, a natural anthraquinone, exhibits cardioprotective effects by specifically inhibiting *P4HB* lactylation at lysine 311, thereby suppressing NDP52-mediated mitophagy and mitigating radiation-induced cardiac injury [118] (Figure 3). However, although studies have focused on the regulation of lactylation, its potential off-target effects need to be carefully discussed. First, both lactylation and acetylation target lysine residues and may share a part of the enzymatic system, and aloin’s intervention in the lactylation pathway may indirectly interfere with the kinetic balance of acetylation modification, for example, through the competitive occupation of lysine sites or by affecting the metabolic availability of cofactors (e.g., NAD+) [119]. Second, the stabilizing effect of aloin on *GOT2*-mediated kynurenine metabolism may influence the microenvironment of other lysine-modified substrates through metabolic reprogramming, thereby affecting hydroxylation or demethylation processes. Although the effect of aloin on acetylation has not been directly reported in the literature, its extensive regulation of mitochondrial functions, ROS metabolism, and inflammatory pathways suggests that its role may go beyond lactylation, and its specificity to other post-translational modifications (e.g., acetylation and ubiquitination) needs to be further tested to fully evaluate its therapeutic potential and risks.

Receptor-Mediated Immunometabolic Modulation: Lactate–GPR81 signaling-axis manipulation offers a novel immunoregulatory strategy. In septic models, lactate-mediated GPR81 activation orchestrates systemic anti-inflammatory responses through the coordinated downregulation of NF-κB-dependent proinflammatory networks, concurrently improving cardiac function and microcirculatory perfusion [120]. This receptor-targeted approach demonstrates the dual advantage of metabolic regulation and immune cell phenotypic modulation.

### 5.3. Future Directions in Lactylation-Targeted Therapeutics

The dynamic nature of lactylation as a metabolic–epigenetic interface presents both opportunities and challenges for next-generation therapeutic development. Three strategic frontiers will likely dominate this field:

The Systems Biology of Lactylation Networks: The metabolic reprogramming of lactylation may comprehensively regulate mitochondrial functions by affecting lactate modification levels of various key enzymes of the TCA cycle and other metabolic pathways, thereby alleviating myocardial ischemia–reperfusion injury to a certain extent and playing important roles in the occurrence and development of metabolic diseases; contrasting evidence shows that *P4HB* lactylation exacerbates radiation-induced cardiotoxicity via dysregulated mitophagy [118] (Figure 2). Such context-dependent effects demand the systematic characterization of the “lactylome” across disease states, integrating metabolomic flux analysis with single-cell epigenetic profiling to identify targetable nodal points.

Chronotherapeutic Modulation Strategies: Future interventions must account for lactylation’s dual temporal dimensions: acute metabolic signaling versus chronic epigenetic remodeling. The development of stimuli-responsive lactylation modulators—activated by disease-specific metabolites or oxidative stress—could enable the dynamic control of this modification. Clinical translation requires addressing three-dimensional heterogeneity: (1) spatial: tissue-specific lactylation thresholds (e.g., cardiac vs. hepatic systems), (2) temporal: stage-dependent modification patterns (early inflammation vs. chronic fibrosis), and (3) individual: genetic/epigenetic variants influencing the lactate metabolic capacity. Emerging technologies, like CRISPR-based lactylation editing and AI-driven multiomic prediction models, could enable personalized regimen design. The case of aloin-mediated *P4HB* lactylation inhibition [118] exemplifies how natural compound libraries may yield selective pharmacophores for specific lactylation sites.

## 6. Conclusions and Future Perspectives

Lactylation has emerged as a pivotal regulatory mechanism that dynamically integrates metabolic flux with epigenetic landscapes, offering unprecedented insights into the pathogenesis of metabolic diseases. Emerging evidence reveals its dual regulatory capacity: transient exercise-induced lactylation enhances insulin sensitivity and mitochondrial plasticity through *AMPK*/*PGC-1α*/*GLUT4* signaling, while chronic hyperlactatemia suppresses mitochondrial biogenesis, driving metabolic diseases through the epigenetic silencing of oxidative metabolism genes. This dichotomy underscores lactylation’s role as a spatiotemporal rheostat, where its effects are contingent upon the metabolic context and tissue-specific thresholds. For instance, hepatic H3K9la promotes HCC development, while a feedback loop between H3K9la and HDAC2 drives VEGF-induced angiogenesis. Such organ specificity highlights the need for precision-targeted interventions to exploit lactylation’s therapeutic potential without disrupting physiological processes, such as neuronal plasticity or immunity surveillance.

Future research should focus on integrating multiomic approaches to clarify lactylation’s spatiotemporal dynamics across organs, especially its tissue-specific thresholds, interactions with metabolism–immunity networks, and hierarchical regulation of metabolic–epigenetic crosstalk, to achieve translational breakthroughs in metabolic disease management.

## Figures and Tables

**Table 1:** Functional description table of MCT subtypes.

Protein	Key Feature(s)	Therapeutic Potential
MCT1	Responsible for lactate and pyruvate transport [26]	Tumor therapeutic target [27]
MCT2	High affinity for lactate and pyruvate	Tumor therapeutic target [28]
MCT3	Expressed in the retinal pigment epithelium and affects lactate transport [29]	Understudied, potential therapeutic targets for retinal diseases
MCT4	Mainly responsible for lactate efflux [30]	Tumor therapeutic target [31]
MCT5	Involved in drug transport	Currently understudied, potential drug delivery system
MCT6	Involved in drug transport	
MCT7	Involved in the outward transport of ketone bodies by hepatocytes during fasting [32]	Potential therapeutic targets for metabolic diseases that are currently understudied
MCT8	Thyroid hormone transporter [33]	Thyroid disease therapeutic targets
MCT9	Outward transporter of carnitine	Understudied
MCT10	Aromatic amino acid transporters	Currently understudied
MCT11	Function not yet defined	Understudied
MCT12	Creatine transporter	Currently understudied
MCT13	Function not yet defined	Understudied
MCT14	Function not yet defined	Currently understudied

## References

[1] Roth G.A., Mensah G.A., Johnson C.O., Addolorato G., Ammirati E., Baddour L.M., Barengo N.C., Beaton A.Z., Benjamin E.J., Benziger C.P. (2020). Global Burden of Cardiovascular Diseases and Risk Factors, 1990–2019. J. Am. Coll. Cardiol..

[2] Brooks G.A. (2018). The Science and Translation of Lactate Shuttle Theory. Cell Metab..

[3] Zhang D., Tang Z., Huang H., Zhou G., Cui C., Weng Y., Liu W., Kim S., Lee S., Perez-Neut M. (2019). Metabolic regulation of gene expression by histone lactylation. Nature.

[4] Wang J., Yang P., Yu T., Gao M., Liu D., Zhang J., Lu C., Chen X., Zhang X., Liu Y. (2022). Lactylation of PKM2 Suppresses Inflammatory Metabolic Adaptation in Pro-inflammatory Macrophages. Int. J. Biol. Sci..

[5] Zhu W., Guo S., Sun J., Zhao Y., Liu C. (2024). Lactate and lactylation in cardiovascular diseases: Current progress and future perspectives. Metabolism.

[6] Haas R., Smith J., Rocher-Ros V., Nadkarni S., Montero-Melendez T., D’acquisto F., Bland E.J., Bombardieri M., Pitzalis C., Perretti M. (2015). Lactate Regulates Metabolic and Pro-inflammatory Circuits in Control of T Cell Migration and Effector Functions. PLOS Biol..

[7] Manoharan I., Prasad P.D., Thangaraju M., Manicassamy S. (2021). Lactate-Dependent Regulation of Immune Responses by Dendritic Cells and Macrophages. Front. Immunol..

[8] Liu X., Li S., Cui Q., Guo B., Ding W., Liu J., Quan L., Li X., Xie P., Jin L. (2024). Activation of GPR81 by lactate drives tumour-induced cachexia. Nat. Metab..

[9] Yang Y., Luo N., Gong Z., Zhou W., Ku Y., Chen Y. (2024). Lactate and lysine lactylation of histone regulate transcription in cancer. Heliyon.

[10] Zhou Y., Liu X., Huang C., Lin D. (2022). Lactate Activates AMPK Remodeling of the Cellular Metabolic Profile and Promotes the Proliferation and Differentiation of C2C12 Myoblasts. Int. J. Mol. Sci..

[11] Theparambil S.M., Kopach O., Braga A., Nizari S., Hosford P.S., Sagi-Kiss V., Hadjihambi A., Konstantinou C., Esteras N., Del Arroyo A.G. (2024). Adenosine signalling to astrocytes coordinates brain metabolism and function. Nature.

[12] Lin Y., Bai M., Wang S., Chen L., Li Z., Li C., Cao P., Chen Y. (2022). Lactate Is a Key Mediator That Links Obesity to Insulin Resistance via Modulating Cytokine Production from Adipose Tissue. Diabetes.

[13] Lagarde D., Jeanson Y., Portais J.-C., Galinier A., Ader I., Casteilla L., Carrière A. (2021). Lactate Fluxes and Plasticity of Adipose Tissues: A Redox Perspective. Front. Physiol..

[14] Sun P., Ma L., Lu Z. (2024). Lactylation: Linking the Warburg effect to DNA damage repair. Cell Metab..

[15] Kambe Y., Kurihara T., Miyata A. (2018). [Astrocyte-neuron lactate shuttle, the major effector of astrocytic PACAP signaling for CNS functions]. Nihon Yakurigaku Zasshi.

[16] Yu X., Zhang R., Wei C., Gao Y., Yu Y., Wang L., Jiang J., Zhang X., Li J., Chen X. (2021). MCT2 overexpression promotes recovery of cognitive function by increasing mitochondrial biogenesis in a rat model of stroke. Anim. Cells Syst..

[17] Chen X., Zhang Y., Wang H., Liu L., Li W., Xie P. (2022). The regulatory effects of lactic acid on neuropsychiatric disorders. Discov. Ment. Health.

[18] Yang C., Pan R.-Y., Guan F., Yuan Z. (2024). Lactate metabolism in neurodegenerative diseases. Neural Regen. Res..

[19] Hagihara H., Shoji H., Otabi H., Toyoda A., Katoh K., Namihira M., Miyakawa T. (2021). Protein lactylation induced by neural excitation. Cell Rep..

[20] Nicholson J.K., Holmes E., Kinross J., Burcelin R., Gibson G., Jia W., Pettersson S. (2012). Host-gut microbiota metabolic interactions. Science.

[21] Zhang J., Xiao Y., Wang H., Zhang H., Chen W., Lu W. (2023). Lactic acid bacteria-derived exopolysaccharide: Formation, immunomodulatory ability, health effects, and structure-function relationship. Microbiol. Res..

[22] Pujada A., Walter L., Patel A., Bui T.A., Zhang Z., Zhang Y., Denning T.L., Garg P. (2017). Matrix metalloproteinase MMP9 maintains epithelial barrier function and preserves mucosal lining in colitis associated cancer. Oncotarget.

[23] Li X., Yang Y., Zhang B., Lin X., Fu X., An Y., Zou Y., Wang J.-X., Wang Z., Yu T. (2022). Lactate metabolism in human health and disease. Signal Transduct. Target. Ther..

[24] Felmlee M.A., Jones R.S., Rodriguez-Cruz V., Follman K.E., Morris M.E. (2020). Monocarboxylate Transporters (SLC16): Function, Regulation, and Role in Health and Disease. Pharmacol. Rev..

[25] Singh M., Afonso J., Sharma D., Gupta R., Kumar V., Rani R., Baltazar F., Kumar V. (2023). Targeting monocarboxylate transporters (MCTs) in cancer: How close are we to the clinics?. Semin. Cancer Biol..

[26] Sun Y., Sun J., He Z., Wang G., Wang Y., Zhao D., Wang Z., Luo C., Tian C., Jiang Q. (2019). Monocarboxylate Transporter 1 in Brain Diseases and Cancers. Curr. Drug Metab..

[27] Huang T., Feng Q., Wang Z., Li W., Sun Z., Wilhelm J., Huang G., Vo T., Sumer B.D., Gao J. (2021). Tumor-Targeted Inhibition of Monocarboxylate Transporter 1 Improves T-Cell Immunotherapy of Solid Tumors. Adv. Healthc Mater..

[28] Alobaidi B., Hashimi S.M., Alqosaibi A.I., Alqurashi N., Alhazmi S. (2023). Targeting the monocarboxylate transporter MCT2 and lactate dehydrogenase A LDHA in cancer cells with FX-11 and AR-C155858 inhibitors. Eur. Rev. Med. Pharmacol. Sci..

[29] Xu J., Zhang Y., Gan R., Liu Z., Deng Y. (2023). Identification and validation of lactate metabolism-related genes in oxygen-induced retinopathy. Sci. Rep..

[30] Zhang L., Xin C., Wang S., Zhuo S., Zhu J., Li Z., Liu Y., Yang L., Chen Y. (2024). Lactate transported by MCT1 plays an active role in promoting mitochondrial biogenesis and enhancing TCA flux in skeletal muscle. Sci. Adv..

[31] Kobayashi M., Narumi K., Furugen A., Iseki K. (2021). Transport function, regulation, and biology of human monocarboxylate transporter 1 (hMCT1) and 4 (hMCT4). Pharmacol. Ther..

[32] Ruppert P.M., Kersten S. (2024). Mechanisms of hepatic fatty acid oxidation and ketogenesis during fasting. Trends Endocrinol. Metab..

[33] Bernal J., Guadaño-Ferraz A., Morte B. (2015). Thyroid hormone transporters—Functions and clinical implications. Nat. Rev. Endocrinol..

[34] Sun S., Li H., Chen J., Qian Q. (2017). Lactic Acid: No Longer an Inert and End-Product of Glycolysis. Physiology.

[35] Hu J., Cai M., Liu Y., Liu B., Xue X., Ji R., Bian X., Lou S. (2020). The roles of GRP81 as a metabolic sensor and inflammatory mediator. J. Cell. Physiol..

[36] Wallenius K., Thalén P., Björkman J.-A., Johannesson P., Wiseman J., Böttcher G., Fjellström O., Oakes N.D. (2017). Involvement of the metabolic sensor GPR81 in cardiovascular control. JCI Insight..

[37] Wang J.-L., Dou X.-D., Cheng J., Gao M.-X., Xu G.-F., Ding W., Ding J.-H., Li Y., Wang S.-H., Ji Z.-W. (2023). Functional screening and rational design of compounds targeting GPR132 to treat diabetes. Nat. Metab..

[38] Krewson E.A., Sanderlin E.J., Marie M.A., Akhtar S.N., Velcicky J., Loetscher P., Yang L.V. (2020). The Proton-Sensing GPR4 Receptor Regulates Paracellular Gap Formation and Permeability of Vascular Endothelial Cells. iScience.

[39] Ren J., Zhang Y., Cai H., Ma H., Zhao D., Zhang X., Li Z., Wang S., Wang J., Liu R. (2016). Human GPR4 and the Notch signaling pathway in endothelial cell tube formation. Mol. Med. Rep..

[40] Chen Z., Wan B., Zhang H., Zhang L., Zhang R., Li L., Zhang Y., Hu C. (2024). Histone lactylation mediated by Fam172a in POMC neurons regulates energy balance. Nat. Commun..

[41] Lee T.Y. (2021). Lactate: A multifunctional signaling molecule. Yeungnam Univ. J. Med..

[42] Yang K., Xu J., Fan M., Tu F., Wang X., Ha T., Williams D.L., Li C. (2020). Lactate Suppresses Macrophage Pro-Inflammatory Response to LPS Stimulation by Inhibition of YAP and NF-kappaB Activation via GPR81-Mediated Signaling. Front. Immunol..

[43] Errea A., Cayet D., Marchetti P., Tang C., Kluza J., Offermanns S., Sirard J.-C., Rumbo M. (2016). Lactate Inhibits the Pro-Inflammatory Response and Metabolic Reprogramming in Murine Macrophages in a GPR81-Independent Manner. PLoS ONE.

[44] Ranganathan P., Shanmugam A., Swafford D., Suryawanshi A., Bhattacharjee P., Hussein M.S., Koni P.A., Prasad P.D., Kurago Z.B., Thangaraju M. (2018). GPR81, a Cell-Surface Receptor for Lactate, Regulates Intestinal Homeostasis and Protects Mice from Experimental Colitis. J. Immunol..

[45] Fang Y., Li Z., Yang L., Li W., Wang Y., Kong Z., Miao J., Chen Y., Bian Y., Zeng L. (2024). Emerging roles of lactate in acute and chronic inflammation. Cell Commun. Signal..

[46] Jiang R., Ren W.-J., Wang L.-Y., Zhang W., Jiang Z.-H., Zhu G.-Y. (2024). Targeting Lactate: An Emerging Strategy for Macrophage Regulation in Chronic Inflammation and Cancer. Biomolecules.

[47] Liu S., Dai Z., Cooper D.E., Kirsch D.G., Locasale J.W. (2020). Quantitative Analysis of the Physiological Contributions of Glucose to the TCA Cycle. Cell Metab..

[48] Jiang C., Ma X., Chen J., Zeng Y., Guo M., Tan X., Wang Y., Wang P., Yan P., Lei Y. (2024). Development of Serum Lactate Level-Based Nomograms for Predicting Diabetic Kidney Disease in Type 2 Diabetes Mellitus Patients. Diabetes Metab. Syndr. Obes..

[49] Maschari D., Saxena G., Law T.D., Walsh E., Campbell M.C., Consitt L.A. (2022). Lactate-induced lactylation in skeletal muscle is associated with insulin resistance in humans. Front. Physiol..

[50] Kjobsted R., Munk-Hansen N., Birk J.B., Foretz M., Viollet B., Björnholm M., Zierath J.R., Treebak J.T., Wojtaszewski J.F. (2017). Enhanced Muscle Insulin Sensitivity After Contraction/Exercise Is Mediated by AMPK. Diabetes.

[51] Knudsen J.R., Steenberg D.E., Hingst J.R., Hodgson L.R., Henriquez-Olguin C., Li Z., Kiens B., Richter E.A., Wojtaszewski J.F., Verkade P. (2020). Prior exercise in humans redistributes intramuscular GLUT4 and enhances insulin-stimulated sarcolemmal and endosomal GLUT4 translocation. Mol. Metab..

[52] Viollet B. (2017). The Energy Sensor AMPK: Adaptations to Exercise, Nutritional and Hormonal Signals.

[53] Hardie D.G. (2022). AMP-activated protein kinase—A journey from 1 to 100 downstream targets. Biochem. J..

[54] Kong S., Cai B., Nie Q. (2022). PGC-1alpha affects skeletal muscle and adipose tissue development by regulating mitochondrial biogenesis. Mol. Genet. Genom..

[55] Yin T.C., Van Vranken J.G., Srivastava D., Mittal A., Buscaglia P., Moore A.E., Verdinez J.A., Graham A.E., Walsh S.A., Acevedo M.A. (2023). Insulin sensitization by small molecules enhancing GLUT4 translocation. Cell Chem. Biol..

[56] Liu S.X., Zheng F., Xie K.-L., Xie M.-R., Jiang L.-J., Cai Y. (2019). Exercise Reduces Insulin Resistance in Type 2 Diabetes Mellitus via Mediating the lncRNA MALAT1/MicroRNA-382-3p/Resistin Axis. Mol. Ther. Nucleic Acids.

[57] Jones T.E., Pories W.J., Houmard J.A., Tanner C.J., Zheng D., Zou K., Coen P.M., Goodpaster B.H., Kraus W.E., Dohm G.L. (2019). Plasma lactate as a marker of metabolic health: Implications of elevated lactate for impairment of aerobic metabolism in the metabolic syndrome. Surgery.

[58] DE-Cleva R., Cardia L., Vieira-Gadducci A., Greve J.M., Santo M.A. (2021). Lactate Can Be a Marker of Metabolic Syndrome in Severe Obesity?. Arq. Bras. Cir. Dig..

[59] Rooney K., Trayhurn P. (2011). Lactate and the GPR81 receptor in metabolic regulation: Implications for adipose tissue function and fatty acid utilisation by muscle during exercise. Br. J. Nutr..

[60] Cai H., Wang X., Zhang Z., Chen J., Wang F., Wang L., Liu J. (2022). Moderate l-lactate administration suppresses adipose tissue macrophage M1 polarization to alleviate obesity-associated insulin resistance. J. Biol. Chem..

[61] Peng X., He Z., Yuan D., Liu Z., Rong P. (2024). Lactic acid: The culprit behind the immunosuppressive microenvironment in hepatocellular carcinoma. Biochim. Biophys. Acta Rev. Cancer.

[62] Godinjak A., Jusufovic S., Rama A., Iglica A., Zvizdic F., Kukuljac A., Tancica I., Rozajac S. (2017). Hyperlactatemia and the Importance of Repeated Lactate Measurements in Critically Ill Patients. Med Arch..

[63] Guo B., Shu H., Luo L., Liu X., Ma Y., Zhang J., Liu Z., Zhang Y., Fu L., Song T. (2023). Lactate Conversion by Lactate Dehydrogenase B Is Involved in Beige Adipocyte Differentiation and Thermogenesis in Mice. Nutrients.

[64] Krycer J.R., Quek L.-E., Francis D., Fazakerley D.J., Elkington S.D., Diaz-Vegas A., Cooke K.C., Weiss F.C., Duan X., Kurdyukov S. (2020). Lactate production is a prioritized feature of adipocyte metabolism. J. Biol. Chem..

[65] Bailey T., Nieto A., McDonald P. (2022). Inhibition of the Monocarboxylate Transporter 1 (MCT1) Promotes 3T3-L1 Adipocyte Proliferation and Enhances Insulin Sensitivity. Int. J. Mol. Sci..

[66] Yao Z., Liang S., Chen J., Zhang H., Chen W., Li H. (2024). Dietary Lactate Intake and Physical Exercise Synergistically Reverse Brown Adipose Tissue Whitening to Ameliorate Diet-Induced Obesity. J. Agric. Food Chem..

[67] Li Z., Liang Z., Qi H., Luo X., Wang M., Du Z., Guo W. (2025). Lactate shuttling links histone lactylation to adult hippocampal neurogenesis in mice. Dev. Cell.

[68] Huang J., Wang X., Li N., Fan W., Li X., Zhou Q., Liu J., Li W., Zhang Z., Liu X. (2024). YY1 Lactylation Aggravates Autoimmune Uveitis by Enhancing Microglial Functions via Inflammatory Genes. Adv. Sci..

[69] Rho H., Terry A.R., Chronis C., Hay N. (2023). Hexokinase 2-mediated gene expression via histone lactylation is required for hepatic stellate cell activation and liver fibrosis. Cell Metab..

[70] Wu S., Li J., Zhan Y. (2024). H3K18 lactylation accelerates liver fibrosis progression through facilitating SOX9 transcription. Exp. Cell Res..

[71] Raju C., Sankaranarayanan K. (2025). Insights on post-translational modifications in fatty liver and fibrosis progression. Biochim. Biophys. Acta Mol. Basis Dis..

[72] Wu H., Huang H., Zhao Y. (2023). Interplay between metabolic reprogramming and post-translational modifications: From glycolysis to lactylation. Front. Immunol..

[73] Yang K., Fan M., Wang X., Xu J., Wang Y., Tu F., Gill P.S., Ha T., Liu L., Williams D.L. (2022). Lactate promotes macrophage HMGB1 lactylation, acetylation, and exosomal release in polymicrobial sepsis. Cell Death Differ..

[74] Liu R., Wu J., Guo H., Yao W., Li S., Lu Y., Jia Y., Liang X., Tang J., Zhang H. (2023). Post-translational modifications of histones: Mechanisms, biological functions, and therapeutic targets. MedComm (2020).

[75] Zhu Z., Zheng X., Zhao P., Chen C., Xu G., Ke X. (2025). Potential of lactylation as a therapeutic target in cancer treatment (Review). Mol. Med. Rep..

[76] Pucino V., Certo M., Bulusu V., Cucchi D., Goldmann K., Pontarini E., Haas R., Smith J., Headland S.E., Blighe K. (2019). Lactate Buildup at the Site of Chronic Inflammation Promotes Disease by Inducing CD4(+) T Cell Metabolic Rewiring. Cell Metab..

[77] Li Y., Cao Q., Hu Y., He B., Cao T., Tang Y., Zhou X.P., Lan X.P., Liu S.Q. (2024). Advances in the interaction of glycolytic reprogramming with lactylation. Biomed. Pharmacother..

[78] Hu X.T., Wu X.-F., Xu J.-Y., Xu X. (2024). Lactate-mediated lactylation in human health and diseases: Progress and remaining challenges. J. Adv. Res..

[79] Lee J.H., Park A., Oh K.-J., Lee S.C., Kim W.K., Bae K.-H. (2019). The Role of Adipose Tissue Mitochondria: Regulation of Mitochondrial Function for the Treatment of Metabolic Diseases. Int. J. Mol. Sci..

[80] Hu Y., He Z., Li Z., Wang Y., Wu N., Sun H., Zhou Z., Hu Q. (2024). Lactylation: The novel histone modification influence on gene expression, protein function, and disease. Clin. Epigenetics.

[81] Mao Y., Zhang J., Zhou Q., He X., Zheng Z., Wei Y., Zhou K., Lin Y., Yu H., Zhang H. (2024). Hypoxia induces mitochondrial protein lactylation to limit oxidative phosphorylation. Cell Res..

[82] Chen Z., Liu M., Li L., Chen L. (2018). Involvement of the Warburg effect in non-tumor diseases processes. J. Cell. Physiol..

[83] Lin J., Ren J. (2024). Lactate-induced lactylation and cardiometabolic diseases: From epigenetic regulation to therapeutics. Biochim. Biophys. Acta Mol. Basis Dis..

[84] Sun W., Jia M., Feng Y., Cheng X. (2023). Lactate is a bridge linking glycolysis and autophagy through lactylation. Autophagy.

[85] Feng F., Wu J., Chi Q., Wang S., Liu W., Yang L., Song G., Pan L., Xu K., Wang C. (2024). Lactylome Analysis Unveils Lactylation-Dependent Mechanisms of Stemness Remodeling in the Liver Cancer Stem Cells. Adv. Sci..

[86] Yao S., Chai H., Tao T., Zhang L., Yang X., Li X., Yi Z., Wang Y., An J., Wen G. (2024). Role of lactate and lactate metabolism in liver diseases (Review). Int. J. Mol. Med..

[87] Xu S., Wu X., Wang S., Xu M., Fang T., Ma X., Chen M., Fu J., Guo J., Tian S. (2024). TRIM56 protects against nonalcoholic fatty liver disease by promoting the degradation of fatty acid synthase. J. Clin. Investig..

[88] Zhou Y., Yan J., Huang H., Liu L., Ren L., Hu J., Jiang X., Zheng Y., Xu L., Zhong F. (2024). The m(6)A reader IGF2BP2 regulates glycolytic metabolism and mediates histone lactylation to enhance hepatic stellate cell activation and liver fibrosis. Cell Death Dis..

[89] Wang N., Wang W., Wang X., Mang G., Chen J., Yan X., Tong Z., Yang Q., Wang M., Chen L. (2022). Histone Lactylation Boosts Reparative Gene Activation Post–Myocardial Infarction. Circ. Res..

[90] Wang Y., Chen L., Zhang M., Li X., Yang X., Huang T., Ban Y., Li Y., Li Q., Zheng Y. (2023). Exercise-induced endothelial Mecp2 lactylation suppresses atherosclerosis via the Ereg/MAPK signalling pathway. Atherosclerosis.

[91] Liao Z., Chen B., Yang T., Zhang W., Mei Z. (2024). Lactylation modification in cardio-cerebral diseases: A state-of-the-art review. Ageing Res. Rev..

[92] Zhang H., Zhao J., Yu J., Zhang X., Ran S., Wang S., Ye W., Luo Z., Li X., Hao Y. (2024). Lactate metabolism and lactylation in cardiovascular disease: Novel mechanisms and therapeutic targets. Front. Cardiovasc. Med..

[93] Raychaudhuri D., Singh P., Chakraborty B., Hennessey M., Tannir A.J., Byregowda S., Natarajan S.M., Trujillo-Ocampo A., Im J.S., Goswami S. (2024). Histone lactylation drives CD8(+) T cell metabolism and function. Nat. Immunol..

[94] Wu Y., Wang Y., Dong Y., Sun L.V., Zheng Y. (2024). Lactate promotes H3K18 lactylation in human neuroectoderm differentiation. Cell. Mol. Life Sci..

[95] Liu J., Wang J., Wang Z., Ren H., Zhang Z., Fu Y., Li L., Shen Z., Li T., Tang S. (2024). PGC-1alpha/LDHA signaling facilitates glycolysis initiation to regulate mechanically induced bone remodeling under inflammatory microenvironment. Bone.

[96] Chen H., Ji X., Lee W.-C., Shi Y., Li B., Abel E.D., Jiang D., Huang W., Long F. (2019). Increased glycolysis mediates Wnt7b-induced bone formation. FASEB J..

[97] Chen J., Zhao D., Wang Y., Liu M., Zhang Y., Feng T., Xiao C., Song H., Miao R., Xu L. (2024). Lactylated Apolipoprotein C-II Induces Immunotherapy Resistance by Promoting Extracellular Lipolysis. Adv. Sci..

[98] Zong Z., Xie F., Wang S., Wu X., Zhang Z., Yang B., Zhou F. (2024). Alanyl-tRNA synthetase, AARS1, is a lactate sensor and lactyltransferase that lactylates p53 and contributes to tumorigenesis. Cell.

[99] Morland C., Andersson K.A., Haugen Ø.P., Hadzic A., Kleppa L., Gille A., Rinholm J.E., Palibrk V., Diget E.H., Kennedy L.H. (2017). Exercise induces cerebral VEGF and angiogenesis via the lactate receptor HCAR1. Nat. Commun..

[100] Yao Z., Liang S., Chen J., Dai Y., Zhang H., Li H., Chen W. (2024). A Combination of Exercise and Yogurt Intake Protects Mice against Obesity by Synergistic Promotion of Adipose Browning. J. Agric. Food Chem..

[101] Chang J.W., Tang C.-H. (2024). The role of macrophage polarization in rheumatoid arthritis and osteoarthritis: Pathogenesis and therapeutic strategies. Int. Immunopharmacol..

[102] Zhao T., Le S., Freitag N., Schumann M., Wang X., Cheng S. (2021). Effect of Chronic Exercise Training on Blood Lactate Metabolism Among Patients with Type 2 Diabetes Mellitus: A Systematic Review and Meta-Analysis. Front. Physiol..

[103] Ren H., Zhang D. (2024). Lactylation constrains OXPHOS under hypoxia. Cell Res..

[104] Sogaard D., Lund M.T., Scheuer C.M., Dehlbæk M.S., Dideriksen S.G., Abildskov C.V., Christensen K.K., Dohlmann T.L., Larsen S., Vigelsø A.H. (2018). High-intensity interval training improves insulin sensitivity in older individuals. Acta Physiol..

[105] Doewes R.I., Gharibian G., Zadeh F.A., Zaman B.A., Vahdat S., Akhavan-Sigari R. (2023). An Updated Systematic Review on the Effects of Aerobic Exercise on Human Blood Lipid Profile. Curr. Probl. Cardiol..

[106] McGee S.L., Hargreaves M. (2024). Exercise performance and health: Role of GLUT4. Free. Radic. Biol. Med..

[107] Scarpelli M.C., Bergamasco J.G.A., Godwin J.S., Mesquita P.H.C., Chaves T.S., Silva D.G., Bittencourt D., Dias N.F., Junior R.A.M., Filho P.C.C. (2024). Resistance training-induced changes in muscle proteolysis and extracellular matrix remodeling biomarkers in the untrained and trained states. Eur. J. Appl. Physiol..

[108] Son W.H., Park H.-T., Jeon B.H., Ha M.-S. (2023). Moderate intensity walking exercises reduce the body mass index and vascular inflammatory factors in postmenopausal women with obesity: A randomized controlled trial. Sci. Rep..

[109] Liu Y., Zhou R., Guo Y., Hu B., Xie L., An Y., Wen J., Liu Z., Zhou M., Kuang W. (2025). Muscle-derived small extracellular vesicles induce liver fibrosis during overtraining. Cell Metab..

[110] Zhao L., Dong M., Ren M., Li C., Zheng H., Gao H. (2018). Metabolomic Analysis Identifies Lactate as an Important Pathogenic Factor in Diabetes-associated Cognitive Decline Rats. Mol. Cell. Proteom..

[111] Yang L., Gilbertsen A., Xia H., Benyumov A., Smith K.A., Herrera J.A., Racila E., Bitterman P.B., Henke C.A. (2023). Hypoxia enhances IPF mesenchymal progenitor cell fibrogenicity via the lactate/GPR81/HIF1alpha pathway. JCI Insight..

[112] Heo J., No M., Cho J., Choi Y., Cho E., Park D., Kim T., Kim C., Seo D.Y., Han J. (2021). Moderate aerobic exercise training ameliorates impairment of mitochondrial function and dynamics in skeletal muscle of high-fat diet-induced obese mice. FASEB J..

[113] Allard N.A., Janssen L., Aussieker T., Stoffels A.A., Rodenburg R.J., Assendelft W.J., Thompson P.D., Snijders T., Hopman M.T., Timmers S. (2021). Moderate Intensity Exercise Training Improves Skeletal Muscle Performance in Symptomatic and Asymptomatic Statin Users. J. Am. Coll Cardiol..

[114] Jing F., Zhu L., Zhang J., Zhou X., Bai J., Li X., Zhang H., Li T. (2024). Multi-omics reveals lactylation-driven regulatory mechanisms promoting tumor progression in oral squamous cell carcinoma. Genome Biol..

[115] Wu D., Spencer C.B., Ortoga L., Zhang H., Miao C. (2024). Histone lactylation-regulated METTL3 promotes ferroptosis via m6A-modification on ACSL4 in sepsis-associated lung injury. Redox Biol..

[116] Zhao X., Li S., Mo Y., Li R., Huang S., Zhang A., Ni X., Dai Q., Wang J. (2021). DCA Protects against Oxidation Injury Attributed to Cerebral Ischemia-Reperfusion by Regulating Glycolysis through PDK2-PDH-Nrf2 Axis. Oxid Med. Cell. Longev..

[117] Skorja M.N., Dolinar K., Miš K., Matkovič U., Bizjak M., Pavlin M., Podbregar M., Pirkmajer S. (2021). Suppression of Pyruvate Dehydrogenase Kinase by Dichloroacetate in Cancer and Skeletal Muscle Cells Is Isoform Specific and Partially Independent of HIF-1?. Int. J. Mol. Sci..

[118] Ouyang F., Li Y., Wang H., Liu X., Tan X., Xie G., Zeng J., Zeng G., Luo Q., Zhou H. (2024). Aloe Emodin Alleviates Radiation-Induced Heart Disease via Blocking P4HB Lactylation and Mitigating Kynurenine Metabolic Disruption. Adv. Sci..

[119] Sun L., Zhang H., Gao P. (2022). Metabolic reprogramming and epigenetic modifications on the path to cancer. Protein Cell.

[120] Madaan A., Nadeau-Vallée M., Rivera J.C., Obari D., Hou X., Sierra E.M., Girard S., Olson D.M., Chemtob S. (2017). Lactate produced during labor modulates uterine inflammation via GPR81 (HCA1). Am. J. Obstet. Gynecol..

